# A multiplex TaqMan qPCR assay for sensitive and rapid detection of phytoplasmas infecting *Rubus* species

**DOI:** 10.1371/journal.pone.0177808

**Published:** 2017-05-17

**Authors:** Holger Linck, Erika Krüger, Annette Reineke

**Affiliations:** 1 Department of Phytomedicine, Hochschule Geisenheim University, Geisenheim, Germany; 2 Department of Pomiculture, Hochschule Geisenheim University, Geisenheim, Germany; Academia Sinica, TAIWAN

## Abstract

Rubus stunt is an economically important disease in the production of raspberries, blackberries, and loganberries. A fast, sensitive, and reliable diagnosis of phytoplasmas, the causal agent of the disease, is of prime importance to stop its spread by vegetative propagation and by insect vectors. Therefore, multiplex qPCR assays using TaqMan probes with different kinds of fluorophores in one reaction were developed, allowing the detection of phytoplasmas in general as well as a more specific detection of phytoplasmas belonging to group 16SrV and host DNA (either plant or insect). This assay now provides a practical tool for the screening of motherplants and monitoring the presence and distribution of phytoplasmas in *Rubus* plants of different geographic origins, cultivars, and cultivation systems, as well as in putative insect vectors like leafhoppers.

## Introduction

Phytoplasmas are cell wall-less plant-pathogenic bacteria that inhabit the phloem of infected plants. They are transferred by phloem feeding insect vectors [1] or by vegetative propagation of infected plants, and can infect more than 700 plant species [2], including many economically important crops. In wild and cultivated *Rubus* species like raspberry (*Rubus ideaeus* L.), blackberry (*Rubus fruticosus* L.), loganberry (*Rubus x loganobaccus*), and European dewberry (*Rubus caesius* L.) they cause a disease referred to as Rubus stunt. Symptoms include stunted growth, shoot proliferation, small leaves, short internodes, enlarged sepals, phyllody, flower proliferation, as well as fruit malformations [3,4].

Rubus stunt is usually associated with *Candidatus* Phytoplasma rubi, which belongs to the 16Sr group of elm yellows phytoplasmas (16SrV) [3,5–9]. However, phytoplasmas from the groups of X-disease (16SrIII), aster yellows (16SrI), and stolbur (16SrXII) have also been identified in Rubus stunt symptomatic *Rubus* spp. plants [9–13], and may cause similar symptoms like the ones described above. So far, little is known about the presence and distribution of phytoplasmas in different *Rubus* species or cultivars, the impact of the respective cultivation system, their geographical spread as well as about the spectrum of putative insect vectors. The only vector of Rubus stunt in raspberries known to date, the leafhopper *Macropsis fuscula* (Hemiptera: Cicadellidae), has been identified in transmission experiments by Fluiter & Meer [14]. As *Rubus* plants are produced by vegetative propagation and the time between plant infection and the development of phytoplasma disease symptoms varies from 4 to 11 months [14], an early detection of phytoplasmas in plant nurseries, using highly sensitive and rapid molecular methods, is of great importance to minimize their future spread.

Because phytoplasma titers in *Rubus* plants are generally very low, regular PCR is often not sensitive enough to detect phytoplasma DNA even in plants with clear proliferation symptoms [15]. Therefore, the most utilized method to acquire diagnostic results is nested PCR [16,17]. Nested PCR is very sensitive, but time consuming, requires post-amplification steps often with hazardous substances [18], and has an increased risk of carry-over contamination [16,19–22]. For the screening of Rubus stunt, qPCR, with its direct and sensitive detection of the amplification product, offers a major advantage compared to nested PCR, due to significant time savings and a reduced risk of false positive results. Furthermore, the possibility to employ TaqMan probes labeled with different fluorogenic dyes enables multiplex detection of different DNA targets in a single reaction tube. Here we present multiplex TaqMan qPCR assays that combine primers and TaqMan probes previously published in literature [23–25] with a newly designed primer and probe pair, allowing a specific, rapid and simultaneous diagnosis of phytoplasma infections in general as well as a more specific detection of elm yellows phytoplasmas (16SrV) infecting *Rubus* species. In addition, DNA of the host (plant or insect) is detected simultaneously in the same assay serving as an internal control.

## Materials and methods

### Plant material and plant DNA extraction

Healthy and Rubus stunt symptomatic raspberry and blackberry plant samples were obtained from different commercial plantings throughout Germany (S1 Table). Plant DNA extraction was done according to a protocol modified from Daire et al. [26]. Leaf or root tissue (1 g) was homogenized in a Bioreba extraction bag <Universal> (Bioreba AG, Switzerland) at room temperature in a mixture of 4 ml of CTAB buffer (3% CTAB, 0.1 M Tris-HCl pH 8.0, 20 mM EDTA, 1.4 M NaCl) and 8 μl of 2-mercaptoethanol. The filtrate was incubated in a water bath at 65°C for 20 min and was extracted with chloroform:isoamyl alcohol (24:1). Nucleic acids were obtained by isopropanol-precipitation. Extracted DNA was dissolved in deionized sterile water and stored at -20°C until use. All DNA extracts (including the insect samples) were measured for the concentration of nucleic acids and protein purity with a NanoDrop ND-1000 spectrophotometer (NanoDrop products, Wilmington, USA).

DNA from the following phytoplasmas was used to check for specificity of the developed assay: Apple proliferation (16SrX-A), aster yellows (16SrI-B), ash yellows (16SrVII-A), and elm yellows (16SrV-A), kindly provided by E. Seemüller (Julius Kühn-Institut, Federal Research Centre for Cultivated Plants, Dossenheim, Germany), Western X (16SrIII-A) and Rubus stunt (16SrV-E), kindly provided by A. Bertaccini (Università di Bologna, Bologna, Italy), and flavescence dorée strain FD70 and palatinate grape vine yellows strain EY17-49 (both 16SrV) kindly provided by M. Maixner (Julius Kühn-Institut, Federal Research Centre for Cultivated Plants, Siebeldingen, Germany). A sample from a symptomless raspberry plant was used as negative control, and deionized sterile water was used as no-template control (NTC).

### Insect samples and insect DNA extraction

Insects present in the canopy of raspberry and blackberry plants were sampled using a G-Vac suction sampler (modified Stihl SH 56, Waiblingen, Germany) in commercial plantings as well as in wild blackberry plants, at different times throughout the growing season. Collections were performed in 2014 at five different locations spread from southern to northern Germany (S2 Table). Phloem sucking insects were sorted, classified according to genus or species if possible, and stored at room temperature in 70% ethanol. DNA extraction was conducted according to a protocol modified from that of Marzachi et al. [27]. Depending on size, one to three insects per extraction were laid-out on tissue paper in order to let the ethanol evaporate. Insects were ground using a micro pestle in a mixture of 500 μl CTAB buffer (2% CTAB, 0.1 M Tris-HCl pH 8.0, 20 mM EDTA, 1.4 M NaCl) and 1 μl 2-mercaptoethanol. After vortexing, the suspension was incubated at 60°C for 30 min and centrifuged for 10 min at 13,000 rpm. The supernatant was transferred to a new tube, extracted with chloroform:isoamyl alcohol (24:1), and DNA was precipitated by adding cold isopropanol. After centrifugation the pellet was washed with 70% ethanol, and dissolved in 20 μl of deionized sterile water. DNA from an individual leafhopper (Hemiptera: Cicadellidae) which was apparently free of phytoplasmas after a PCR with primer pairs P1/P7 (see below) was used as a negative control. Deionized sterile water was used as no-template control.

### Oligonucleotide primers and probes

**Fig 1:**
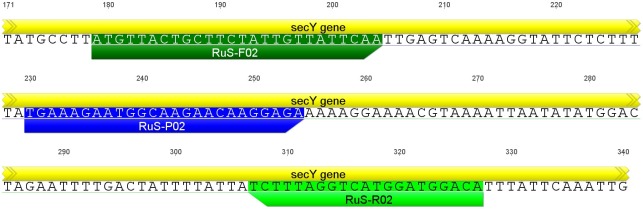
Sequence of the *secY* gene of *Ca*. phytoplasma rubi used for group specific amplification and detection of elm yellow phytoplasmas. Locations of forward primer (RuS-F02), probe (RuS-P02), and reverse primer (RuS-R02) are indicated. Numbering of nucleotide positions is according to GenBank accession number AM397299.

Elm yellows group specific primers and TaqMan probes were designed for the *secY* gene of *Ca*. Phytoplasma rubi (GenBank accession number AM397299) (Fig 1) using PrimerQuest (Integrated DNA Technologies, Inc., Coralville, Iowa, USA). Therefore, we aligned 14 sequences of *secY* genes of different phytoplasmas using Geneious 6.1.7 (Biomatters Ltd., Auckland, New Zealand). Respective accession numbers and origins are provided in S3 Table. Specificity was checked by using NCBI’s Primer-BLAST for the primers and Nucleotide BLAST for the TaqMan probe (https://blast.ncbi.nlm.nih.gov/Blast.cgi). Furthermore, Geneious 6.1.7 (Biomatters Ltd., Auckland, New Zealand) was used to create an alignment of phytoplasma strains used to test for specificity in the validation of the assay for plant material, showing primer binding sites (S1 Fig). For the universal detection of phytoplasmas a primer and probe pair from Christensen et al. [23] was used. In addition, a primer and probe set for detection of host plant DNA [24] and for host insect DNA [25], both targeting the 18S rDNA, were used as an internal control. TaqMan probes were either labelled with FAM, ROX, or Cy5, allowing simultaneous detection of three targets in a single reaction. Sequences, expected size of the amplification product, specificity, final concentrations, and fluorogenic dyes used for each primer and probe combination are shown in Table 1. All oligonucleotide primers and probes were synthesized by Biolegio (Nijmegen, the Netherlands).

**Table 1:** Sequence, size of the expected PCR product, specificity, final concentration, and attached fluorophores for primers and probes used in the Rubus stunt multiplex qPCR assay.

Primer /Probe	Sequence (5’– 3’)	Specificity	Final Concentration (nM)		Fluorophore	Reference
			Plant Assay	Insect Assay		
RuS-F02	ATGTTACTGCTTCTATTGTTATTCAA	16SrV	400	200	-	This study
RuS-R02	TGTCCATCCATGACCTAAAGA		400	200	-	
RuS-P02	TGAAAGAATGGCAAGAACAAGGAGA		400	200	FAM	
UPPFw*	CGTACGCAAGTATGAAACTTAAAGGA	phytoplasma universal	100	100	-	[23]
UPPRv*	TCTTCGAATTAAACAACATGATCCA		100	100	-	
UPPProbe*	TGACGGGACTCCGCACAAGCG		100	100	Cy5	
18SF*	AGAGGGAGCCTGAGAAACGG	plant host DNA	100	-	-	[24]
18SR*	CAGACTCATAGAGCCCGGTATTG		100	-	-	
18SP*	CCACATCCAAGGAAGGCAGCAGGCG		100	-	ROX	
MqFw	AACGGCTACCACATCCAAGG	insect host DNA	-	100	-	[25]
MqRv	GCCTCGGATGAGTCCCG		-	100	-	
MqProbe	AGGCAGCAGGCACGCAAATTACCC		-	100	ROX	

*as designated by the authors of the paper on hand

### Standard curve

To generate a qPCR standard curve for the elm yellows phytoplasma specific primers and probe a PCR with primer pairs RuS-F02 and RuS-R02 was carried out with *Ca*. Phytoplasma rubi DNA to get a 149 bp amplicon. The amplicon was purified with the Hi Yield Gel/PCR DNA Fragment Extraction Kit (Süd-Laborbedarf GmbH, Gauting, Germany) and cloned using the pGEM-T Easy Vector System II (Promega GmbH, Mannheim, Germany). The purified plasmids were quantified with a NanoDrop ND-1000 spectrophotometer (NanoDrop products, Wilmington, USA). The number of molecules in one μl of plasmid solution was calculated based on the molecular weight using the formula: number of copies = plasmid concentration/[(plasmid size + insert (bp) × 660)/(Avogadros’s number)]. In this case, the purified cloned pGEM-T Easy Vector (3015 + 149 bp) had a concentration of 1.24 × 10^−7^ g/μl. Hence, the number of copies was 3.59 × 10^10^ molecules/μl. Ten-fold serial dilutions from 1 × 10^9^ to 1 × 10^5^ molecules/μl of the purified plasmids were used to generate the standard curve. As the *secY* gene is a single copy gene in phytoplasma genomes [28] the copy number corresponds to the number of phytoplasma cells.

In addition, the same serial dilution of plasmid DNA was run with 1 μl of a DNA extract (at 100 ng/μl nucleic acid) from an uninfected raspberry plant on the same qPCR plate as the standard curve, to evaluate the degree of interference of plant DNA extracts in the quantification of phytoplasma copy numbers. All samples were run in triplicate.

### TaqMan qPCR assay

Oligonucleotides were combined in a qPCR assay to detect DNA of elm yellows phytoplasmas (RuS-F02, RuS-R02, RuS-P02), phytoplasma DNA in general (UPPFw, UPPRv, UPPProbe), and either plant (18SF, 18SR, 18SP) or insect (MqFw, MqRv, MqProbe) host DNA as an internal control. Assays were run in 25 μl reactions using the KAPA PROBE FAST Master Mix (2X) Universal (Kapa Biosystems, Cape Town, South Africa) on an iQ5 real-time thermal cycler (Bio-Rad Laboratories, Hercules, CA, USA), with an initial denaturation step of 20 sec at 95°C followed by 40 cycles with 3 sec denaturation at 95°C and 30 sec annealing and elongation at 60°C. Optimal final concentrations of primer and probe pairs were initially determined empirically and are shown in Table 1.

The multiplex assays were validated by comparing quantification cycle (C_q_) values of samples that were run both in singleplex and multiplex assays on the same 96-well plate. One validation assay was run for plant samples and one for insects. For the plant assay DNA from a Rubus stunt symptomatic infected raspberry plant was adjusted to 250 ng/μl of total nucleic acid and then used in six 10-fold (from 1 to 10^−5^) serial dilutions. Since there was no insect sample with DNA from *Ca*. Phytoplasma rubi available, an artificial reference sample was created by mixing DNA from a phytoplasma-free leafhopper (same sample as the one used as a negative control in the assays) with *Ca*. Phytoplasma rubi DNA from an infected periwinkle (*Catharanthus roseus*) plant in equal parts. This sample was adjusted to 200 ng/μl of total nucleic acid and was used in the same serial dilutions as the plant DNA sample. All samples were run in triplicate in qPCR assays with 2 μl of DNA solution.

The assay for plant material was further validated by mixing fresh leaf material of infected and healthy raspberry plants in known weight ratios in a total of 1 g, to obtain proportions of 100, 75, 50, 33, 11, 6, 1, and 0% infected leaf material, respectively. Each mixture was extracted in 3 independent replicates. These samples were diluted to a concentration of 100 ng/μl of total nucleic acid for use in the qPCR assay and were run in triplicate as described above.

### Nested PCR

Nested PCR was run with primer pairs P1/P7 [29, 30] for the direct PCR, followed by U5/U3 [31] for the nested PCR. DreamTaq DNA Polymerase (Thermo Fisher Scientific, Waltham, MA, USA) was used in 20 μl reactions on a MyCycler Thermal Cycler System (Bio-Rad Laboratories, Hercules, CA, USA). The product of the direct PCR was diluted 1:30 in deionized sterile water for use in the nested PCR. For comparability with the multiplex assay validation, the same serial dilutions as in the multiplex validation assays were run in nested PCRs. PCR products were visualized on 1% agarose gels stained with Invitrogen SYBR Safe DNA Gel Stain (Thermo Fisher Scientific, Waltham, MA, USA) and were run for 60 min at 80 V.

## Results

### Standard curve

**Fig 2:**
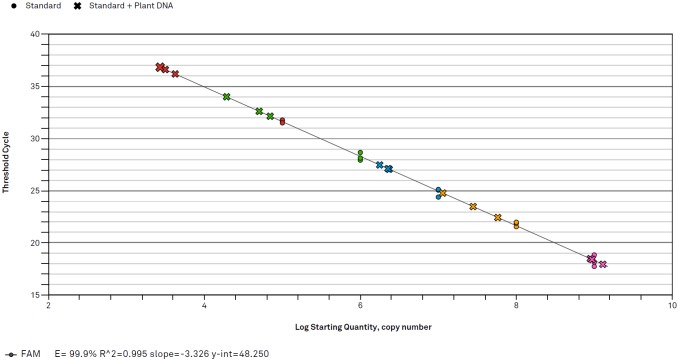
Standard curve for the elm yellows specific primers and probe of the multiplex TaqMan qPCR assay. The standard curve was generated with 10-fold dilutions of plasmid DNA (Standard, indicated by a dot) containing an insert from the *secY* gene of *Ca*. Phytoplasma rubi. In addition, plasmid DNA was mixed with raspberry plant DNA (Standard + Plant DNA, indicated by a cross) to show interference of the DNA extract when quantifying phytoplasmas in plant tissues.

The efficiency of the Multiplex TaqMan qPCR assay for the elm yellows specific primers and TaqMan probe presented by the standard curve using plasmid DNA containing the cloned phytoplasma amplicon was 99.9% (Fig 2). For target concentrations of at least 1 × 10^9^ copy numbers, obtained C_q_-values were similar for pure plasmid DNA and a mixture of plasmid DNA with plant DNA. Accordingly, 1 × 10^9^ copy numbers can be regarded as a necessary threshold value for accurate quantification of phytoplasma DNA in infected plant tissues.

### Validation of the multiplex TaqMan assay for plant material

**Fig 3:**
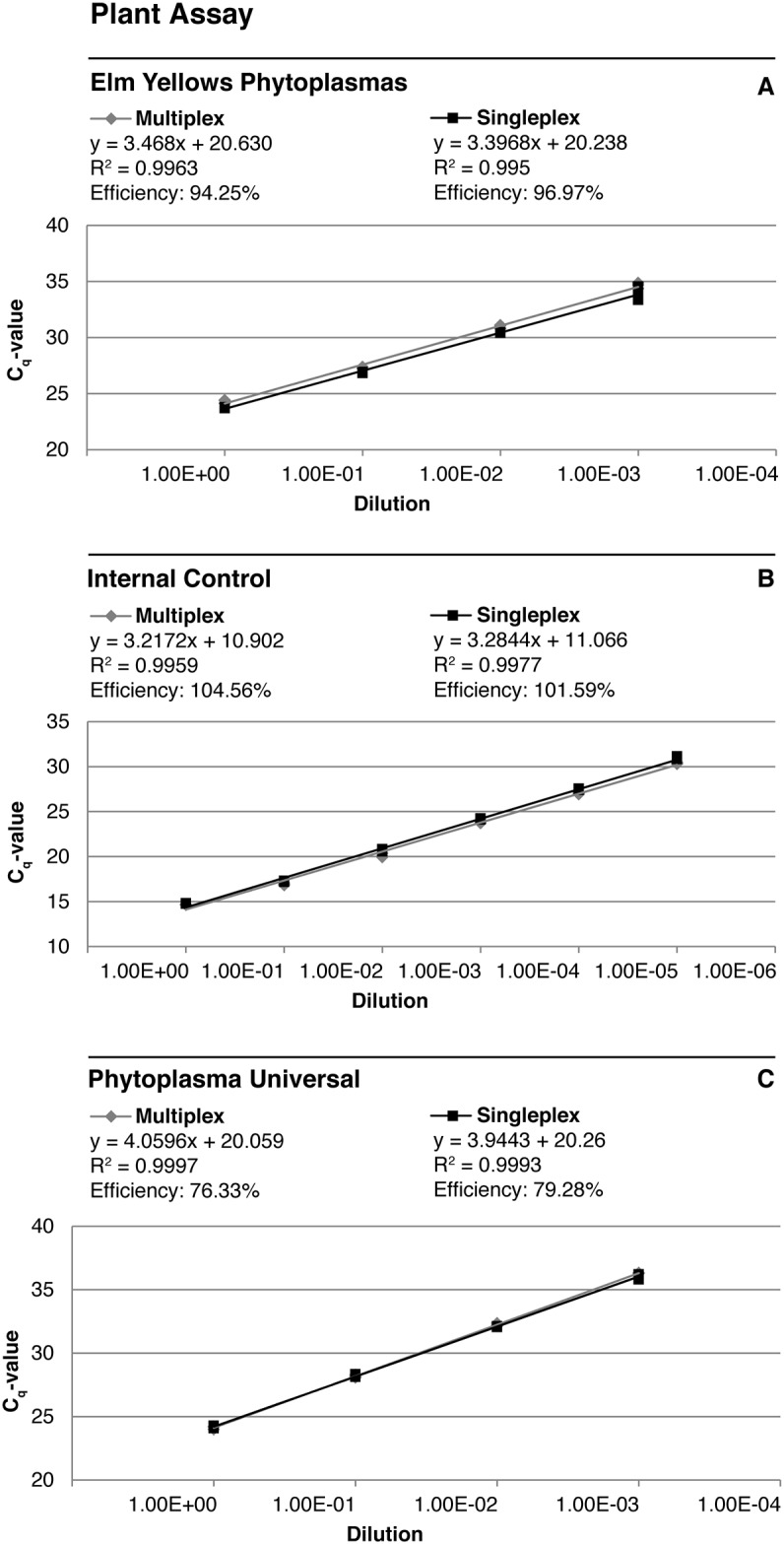
Standard curves obtained in the multiplex validation assay with *Rubus* plant samples. C_q_-values of the singleplex and multiplex TaqMan qPCR are plotted against a 10-fold serial dilution from a Rubus stunt positive DNA extract with primers and probes for the detection of (A) elm yellows phytoplasmas, (B) plant host DNA as an internal control, and (C) phytoplasmas in general. Slopes, R^2^ and efficiencies of the respective reactions are presented for each curve.

The results of the multiplex TaqMan qPCR assay validation on *Rubus* plants infected with phytoplasmas are shown in Fig 3. Graphs show C_q_-values plotted against serial dilutions from a Rubus stunt infected raspberry DNA extract. Both primer/probe sets for the detection of phytoplasma DNA (elm yellows phytoplasmas and phytoplasma universal) were able to detect their respective targets along a 10-fold serial dilution gradient from undiluted DNA (500 ng of total nucleic acid) up to a dilution of 10^−3^. The internal control for plant host DNA yielded positive results for all six 10-fold serial dilutions. There were no obvious differences in C_q_-values between multiplex and singleplex assays (Fig 3). The negative control showed a signal only for the internal control (plant host DNA). No signals were obtained for the no-template controls (data not shown).

**Fig 4:**
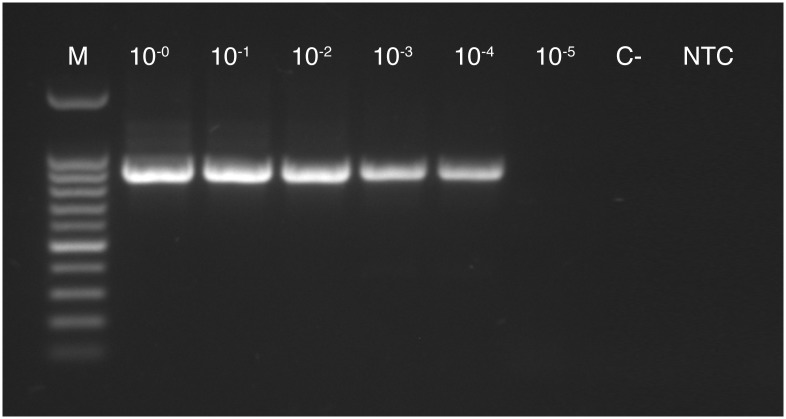
Phytoplasma specific nested PCR products of a 10-fold serial dilution (10^0^ to 10^−5^) from a Rubus stunt positive DNA extract using primer pairs P1/P7 and U5/U3. For nested PCR the same DNA extracts as in the validation assay for the multiplex qPCR for plant samples (Fig 3) were used. (M) Metabion mi-100 bp+ DNA Marker Go, (C-) negative control, (NTC) no-template control.

When the same samples were run in nested PCR with primer pairs P1/P7 (direct PCR) and U5/U3 (nested PCR), an amplification product of the expected size could be obtained up to a dilution of 10^−4^ (Fig 4). However, the nested PCR had to be carried out 4 times and had to be set up under an UV sterilization cabinet in order to achieve results without contaminations in the no-template control or the negative control.

For routine purposes in diagnostics of phytoplasmas, material of several asymptomatic plants (whether infected or not) is often mixed, resulting in different amounts of phytoplasma infected material. When the qPCR assay for plant samples was run with different proportions of infected and healthy leaf material (100, 75, 50, 33, 11, 6, 1, and 0% infected material), it was evident that it is possible to reliably detect phytoplasma DNA in samples containing only 1% of infected leaf material (S4 Table).

When the assay was run with phytoplasma DNA from aster yellows (16SrI-B), ash yellows (16SrVII-A), Western X (16SrIII-A), elm yellows (16SrV-A), palatinate grapevine yellows (16SrV), flavescence dorée (16SrV), Rubus stunt (16SrV-E), and apple proliferation (16SrX-A), all were positive for phytoplasmas in general. A positive signal for the 16SrV specific primer-probe combination was only obtained for elm yellows, palatinate grapevine yellows, flavescence dorée, and Rubus stunt DNA (Table 2).

**Table 2:** Results from the developed multiplex TaqMan qPCR assay when run with strains from a variety of different phytoplasma groups. Mean C_q_-values of 3 technical replicates are shown. C_q_-values below 38 are regarded as positive values. (N/A) not applicable because no fluorescent signal above the background fluorescence could be detected.

Phytoplasma strain	Plant host DNA	16SrV Phytoplasmas	Phytoplasma universal
aster yellows (16SrI-B)	13.59	N/A	18.32
Western X (16SrIII-A)	33.29	N/A	34.52
elm yellows (16SrV-A)	13.96	18.59	17.69
palatinate grapevine yellows strain EY17-49 (16SrV)	13.01	19.39	16.59
flavescence dorée strain FD70 (16SrV)	14.27	21.95	18.31
Rubus stunt (16SrV-E)	22.27	28.47	27.32
ash yellows (16SrVII-A)	13.68	N/A	15.85
apple proliferation (16SrX-A)	13.73	N/A	19.22

### Validation of the multiplex TaqMan assay for phytoplasmas present in insects

**Fig 5:**
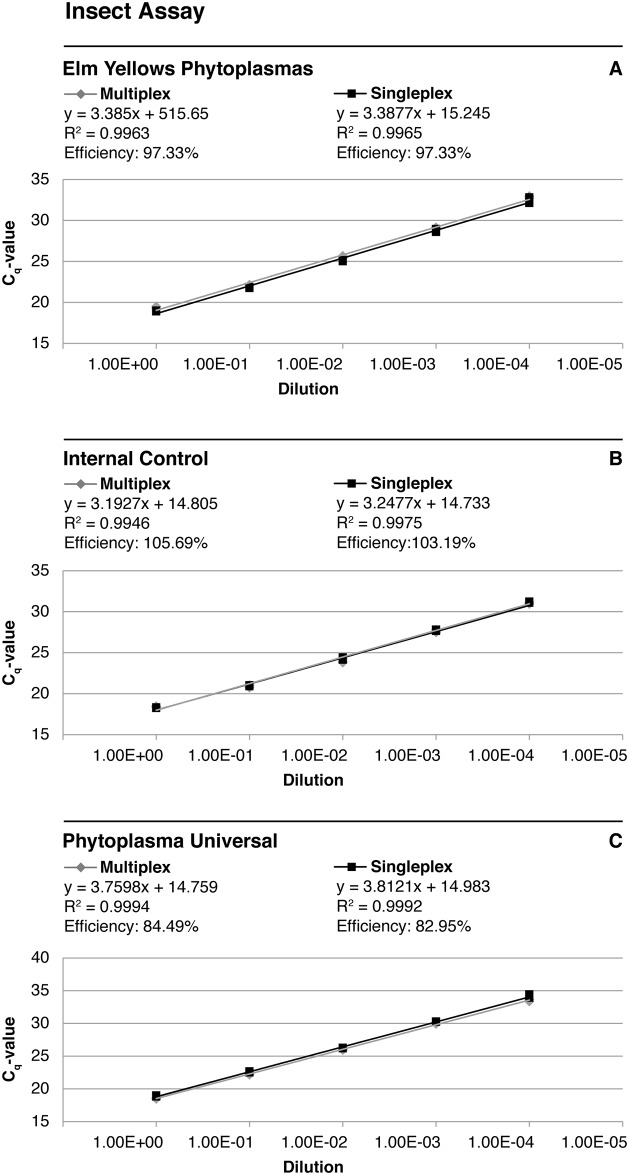
Standard curves obtained in the multiplex validation assay with insect samples. C_q_-values of the singleplex and multiplex TaqMan qPCR are plotted against a 10-fold serial dilution of a DNA mixture from an uninfected leafhopper sample and *Catharanthus roseus* infected with *Ca*. Phytoplasma rubi. Primers and probes for detection of (A) elm yellows phytoplasmas, (B) insect host DNA as an internal control, and (C) phytoplasmas in general were used. Slopes, R^2^ and efficiencies of the respective reactions are presented for each curve.

When the multiplex TaqMan assay was applied to insect DNA artificially mixed with phytoplasma DNA, all respective targets (elm yellow phytoplasma DNA, phytoplasma DNA in general, and insect host DNA) could be detected up to a serial DNA dilution of 10^−4^ (Fig 5). C_q_-values did not differ from each other when comparing multiplex with singleplex assays. All no-template controls were negative, and the negative control was positive only for the internal control (data not shown).

**Fig 6:**
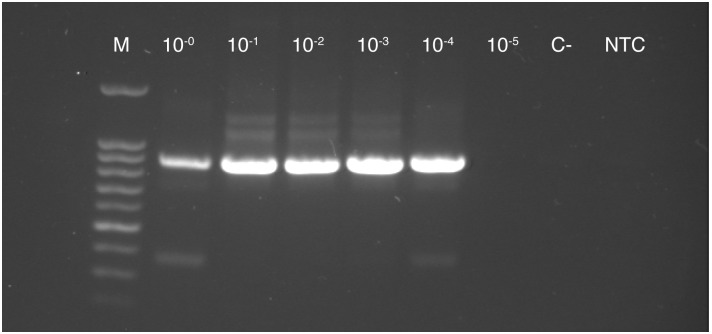
Phytoplasma specific nested PCR products of a 10-fold serial dilution (10^0^ to 10^−5^) of a DNA mixture containing DNA of an uninfected leafhopper and *Catharanthus roseus* infected with *Ca*. Phytoplasma rubi using primer pairs P1/P7 and U5/U3. For nested PCRs the same DNA sample as in the validation assay for multiplex qPCR for insect samples (Fig 4) were used. (M) Metabion mi-100 bp+ DNA Marker Go, (C-) negative control, (NTC) no template control.

Nested PCR was able to detect phytoplasma DNA in insect DNA samples up to a dilution of 10^−4^, the same dilution as the multiplex TaqMan qPCR assay (Fig 6).

### Field validation of the multiplex TaqMan assay

A total of 140 raspberry and blackberry plant DNA samples (leaf, roots, bast, peduncle and fruit) obtained from 9 different commercial plantings throughout Germany were tested for the presence of phytoplasmas using both the newly developed multiplex TaqMan qPCR assay and the standard nested PCR assay. Some of the plants were showing distinctive symptoms pointing to a phytoplasma infection, however, for the majority of plants, these symptoms were not clearly visible (S1 Table). Of these samples, 85 were positive for phytoplasma DNA with the multiplex TaqMan qPCR assay whereas only 82 samples were positive with nested PCR. The qPCR assay was positive in 6 cases where nested PCR was negative and, in turn, nested PCR was positive in 3 cases where the qPCR was negative (S1 Table). Again, nested PCRs had to be repeated several times and had to be set up under sterile conditions to obtain amplification-free no-template controls or negative controls.

In the course of monitoring putative Rubus stunt insect vectors in five different commercial raspberry and blackberry fields throughout Germany in 2014, 1293 individual phloem sucking insects were caught on raspberry and blackberry plants. After sorting and classification, 379 DNA samples of putative Rubus stunt insect vectors representing the full range of sampled locations, dates, and phloem sucking insects were extracted and analysed with the multiplex TaqMan qPCR assay as described above. Nine DNA samples were tested positive for the presence of phytoplasmas in general, but negative for Elm yellows phytoplasmas (S2 Table). Three of these phytoplasma positive samples were from leafhoppers of the genus *Euscelidius* and six from leafhopper species identified as *Macrosteles* spp.

## Discussion

Phytoplasmas are known as serious pathogens of a variety of commercial crop plants, with their diagnosis often being challenging due to unspecific symptoms, a long period of latency and low concentrations in sample tissues because they are limited to the phloem. Therefore, a fast, sensitive, and reliable diagnostic method is of prime importance to minimize their spread by insect vectors and by vegetative propagation. Nested PCR has been the most commonly used tool for the detection of phytoplasmas since the early 1990s [17,32]. However, nested PCR has a high risk for cross-contaminations and needs time consuming post-amplification steps. We know of 28 publications to date in which qPCR assays for detection of phytoplasmas were developed (S5 Table). Of these 28 papers, 8 employed DNA dyes (SYBR Green or EvaGreen), whereas 21 used TaqMan probes (one paper used both, SYBR Green and TaqMan chemistry, in separate assays). Despite of this high number of published TaqMan assays for phytoplasma detection only 7 papers employed a multiplex approach. These 7 assays combine the following specificities: apple proliferation and *Malus domestica* [33]; aster yellows (16SrI) or other group [34]; flavescence dorée (16SrV), bois noir (16SrXII-A), and grapevine [20]; stolbur (16SrXII-A), *Candidatus* Phlomobacter fragariae, and plant DNA [35]; phytoplasmas in general and plant host DNA [24]; phytoplasmas in general, pear blister canker viroid, and apple scar skin viroid [36]; European stone fruit yellows (16SrX-B) and plant host DNA [37]. Here, we present a multiplex phytoplasma TaqMan qPCR assay that allows for the first time a fast and simultaneous detection of phytoplasmas in general, a group specific detection of elm yellows phytoplasmas (16SrV), and the detection of either host plant DNA or insect vector DNA in one single reaction. TaqMan assays for detection of phytoplasmas were shown to be at least as sensitive as nested PCR [21,30,38], but less susceptible to inhibitory substances in the reaction mixture [24]. Accordingly, DNA extracts can be used less diluted for TaqMan assays, usually resulting in higher detection sensitivities compared to nested PCR assays. However, this well-known advantage of TaqMan assays could not be proven in this study as both the multiplex TaqMan qPCR assay and nested PCR were able to detect the highest concentration levels of the tested serial dilutions of target DNAs. When comparing the multiplex TaqMan qPCR assay for plant material with results from nested PCR, the nested PCR was able to show a clear PCR product for one dilution factor higher than qPCR. However, labor-intensive nested PCR is not suitable for routine analysis in plant nurseries where usually high numbers of samples need to be analyzed as quickly and accurate as possible. In addition, we needed to set up nested PCRs under an UV sterilization cabinet in order to achieve reproducible results with clean negative controls and/or no-template controls. Unspecific PCR products and false positive or negative results are a general problem of nested PCR due to its high sensitivity combined with high risks for contaminations [16,37]. Moreover, we assume that the discrepancies in results obtained via qPCR and nested PCR with the same raspberry DNA sample are due to a limit of detection and/or the presence of inhibitory substances in the DNA extract.

The possibility of multiplexing by equipping TaqMan probes with different fluorogenic dyes allows detecting multiple targets in a single reaction. In the assays presented here, multiplexing was used not only to detect specifically elm yellows phytoplasmas and phytoplasmas in general at the same time, but also to include internal controls detecting either insect or plant host DNA. This enables the confirmation of a successful DNA extraction, and excludes false negative results resulting from a potential inhibition of the PCR. This, together with the lower risk of contamination due to detection of amplification products in a closed-tube system, makes the assays much more reliable than nested PCR. Furthermore, multiplex TaqMan assays are more time-saving than nested PCR because there is no need for post-PCR processing, and only one round of PCR has to be carried out. Using the KAPA PROBE FAST Master Mix as described here, once PCR reactions are fully set up on a 96-well plate, about 1 hour is needed until results can be obtained. In comparison, with nested PCR approximately about 6–8 hours are needed to get results from the same number of samples. In addition, the results obtained here using different proportions of phytoplasma infected and healthy leaf material showed that it can be a valid option for plant nurseries to pool multiple leaf samples of different plants while still being able to reliably detect phytoplasmas and thus saving even more time and resources. Accordingly, the assays developed in this study provide a rapid and practical tool for screening of *Rubus* motherplants for the presence of both elm yellows phytoplasmas (to which Rubus stunt *Ca*. Phytoplasma rubi belongs) and phytoplasmas in general in nurseries or during plant propagation.

Screening of putative insect vectors showed that leafhoppers of the genus *Euscelidius* and *Macrosteles* who apparently had previously sucked on phytoplasma infected plants were present in commercial *Rubus* plantings. Both species are known as vectors of phytoplasmas [39,40], however, it remains to be shown in insect transmission assays if these leafhoppers are in fact able to also transmit Rubus stunt phytoplasmas to *Rubus* species. In any case, the multiplex qPCR TaqMan assay developed in the present study will now allow a quick and reliable identification of phytoplasma insect vectors in orchards. Since measurements targeting insect vectors of phytoplasmas are the only options for managing phytoplasmas and particularly for preventing their spread within and among orchards, a detailed knowledge of respective vectors is a prerequisite for improvement of plant protection strategies against phytoplasma diseases.

## Supporting information

S1 Fig Sequence alignment of the phytoplasma strains used for testing specificity shown in Table 2 (except for palatinate grapevine yellows, as there was no sequence available), showing the binding sites of RuS-F02, RuS-P02, and RuS-R02. (AM397299) Rubus stunt 16SrV-E, (AY197686) flavescence dorée 16SrV, (AY197690) elm yellows 16SrV-A, (GU004329) ash yellows 16SrVII-A, (GU004354) Western X 16SrIII-A, (GU004335) apple proliferation 16SrX-A, and (AY803177) aster yellows 16SrI-B.
(TIF)

S1 Table Raspberry plant samples. Locations used for sampling as well as tissue types are presented. The same DNA extract was used for qPCR and nested PCR analysis.
(XLSX)

S2 Table Sampled putative insect vectors. Individual insect samples with positive qPCR results for the presence of phytoplasmas are shaded in grey.
(XLSX)

S3 Table Accession numbers used in sequence alignment of *secY* genes of different phytoplasmas. (XLSX)

S4 Table Mean Cq-values and standard deviations of the multiplex TaqMan qPCR assay for DNA samples obtained from different proportions of phytoplasma infected and healthy leaf material. Each leaf sample was extracted in 3 independent replicates (designated as 1–3) and each DNA extract was analysed in triplicate in the qPCR.
(XLSX)

S5 Table Publications in which qPCR assays for the detection of phytoplasmas were developed. (XLSX)
