# Supplementary figures and images for: A multiplex TaqMan qPCR assay for sensitive and rapid detection of phytoplasmas infecting Rubus species
Source: PLoS One. 2017 May 17;12(5):e0177808. doi: 10.1371/journal.pone.0177808 (PMC5435344; doi:10.1371/journal.pone.0177808)

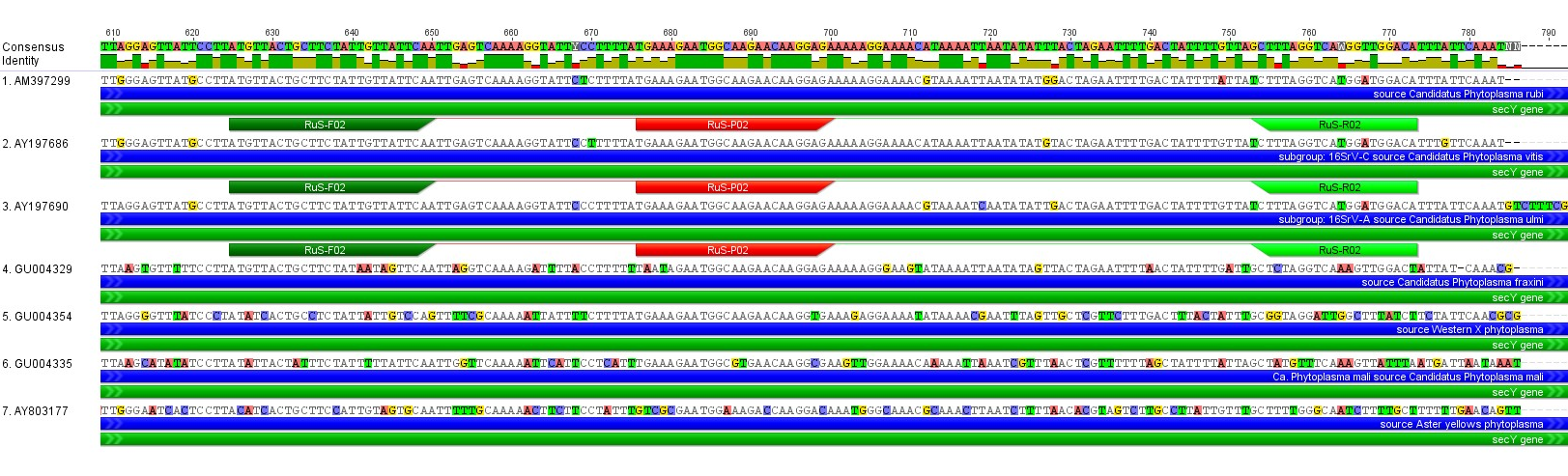

Supplement: S1 Fig — (AM397299) Rubus stunt 16SrV-E, (AY197686) flavescence dorée 16SrV, (AY197690) elm yellows 16SrV-A, (GU004329) ash yellows 16SrVII-A, (GU004354) Western X 16SrIII-A, (GU004335) apple proliferation 16SrX-A, and (AY803177) aster yellows 16SrI-B. (TIF) [file pone.0177808.s001.tif]
